# Adjusting primary-care funding by deprivation: a cross-sectional study of Lower layer Super Output Areas in England

**DOI:** 10.3399/BJGPO.2024.0185

**Published:** 2025-01-29

**Authors:** Ian Holdroyd, Cameron Appel, Efthalia Massou, John Ford

**Affiliations:** 1 Wolfson Institute of Population Health and Primary Care, Queen Mary University of London, London, UK; 2 Department of Public Health and Primary Care, University of Cambridge, Cambridge, UK

**Keywords:** inequalities, health inequities, general practice, primary healthcare

## Abstract

**Background:**

Previous research has called for general practice funding to be adjusted by deprivation data. However, there is no evidence that this adjustment would better meet clinical need.

**Aim:**

To assess (1) how accurately the capitation formula (Carr-Hill), and total general practice funding predicts clinical need and (2) whether adjusting by the Index of Multiple Deprivation (IMD) score improves accuracy.

**Design & setting:**

A cross-sectional analysis of 32 844 Lower layer Super Output Areas (LSOAs) in England in 2021–2022. Sensitivity analysis used data from 2015–2019.

**Method:**

Weighted average Carr-Hill Index (CHI), total general practice funding, and five measures of clinical need were calculated for each LSOA. For both CHI and total funding, four sets of generalised linear models were calculated for each outcome measure: unadjusted; adjusted for age; adjusted for IMD; and adjusted for age and IMD. Adjusted *R* ^2^ assessed model accuracy.

**Results:**

In unadjusted models, CHI was a better predictor than total funding of combined morbidity index (CMI) (*R* ^2^ = 49.81%, 29.31%, respectively), combined diagnosed and undiagnosed morbidity (*R* ^2^ = 43.52%, 21.39%) and emergency admissions (*R* ^2^ = 32.75%, 16.95%). Total funding was a better predictor than CHI of GP appointments per patient (*R* ^2^ = 28.5%, 22.5%, respectively) and age and sex standardised mortality rates (*R* ^2^ = 0.42%, 0.37%). Adjusting for age and IMD improved all 10 models (*R* ^2^ = 62.15%, 53.15%, 48.57%, 38.47%, 40.53%, 32.84%, 29.11%, 34.58%, 25.21%, 25.23%, respectively). All age and IMD adjusted models significantly outperformed age-adjusted models (*P*<0.001). Sensitivity analysis confirmed findings.

**Conclusion:**

Adjusting capitation or total funding by IMD would increase funding efficiency, especially for long-term outcomes such as mortality. However, adjusting for IMD without age could have unwanted consequences.

## How this fits in

Much evidence highlights how primary care funding in the UK exacerbates existing health inequalities. Many have suggested that primary care funding should be adjusted for deprivation. There is currently no evidence on the likely effect of such an adjustment. This study provides crucial insights into the effects of such an adjustment, in addition to unintended consequences and how these can be mitigated.

## Introduction

Ensuring resourcing of primary care proportionate to need is paramount to delivering cost-effective care across the entire healthcare system. In the UK, approximately half of general practice funding follows a capitation model, where patient demographics drive the calculation of clinical need. This model, commonly known as the Carr-Hill formula, includes age, sex, morbidity, mortality, list turnover, nursing and residential homes, geographical staff cost differences, and rurality.^1^ The outcome is a Carr-Hill Index (CHI) for each practice, which is multiplied by the number of registered patients to determine the total number of weighted patients. In 2023, for each weighted patient, the practice received £102.28.^2^ General practices also receive income through a range of other mechanisms, such as additional services (for example, vaccinations or prescribing), performance-based financial incentives, research and teaching.^3–5^

Critics argue that the Carr-Hill formula, which is more than 20 years old, is outdated and fails to adequately measure clinical need.^3,6^ Previous research has found that a 10% increase in the practice Index of Multiple Deprivation (IMD) score correlates with a mere 0.06% increase in practice payments.^7^ This small increase in funding is in contrast to higher clinical need, and worse clinical outcomes in areas with greater deprivation scores.^7^ Arguably areas with higher deprivation scores are comparatively underfunded, compared with more affluent areas with lower clinical need.^4,6^

Previous research has recommended adjusting primary care funding to include an area’s socioeconomic status.^6,7^ Such an approach is used effectively internationally.^8^ However, we lack evidence demonstrating that its inclusion would yield a more accurate measure of clinical need; nor is there sufficient evidence exploring potential unwanted consequences of such a change. One potential cause of an unwanted consequence is that the effects of socioeconomic status may be overshadowed by age, and more affluent areas have older populations, which could result in funding being diverted to more affluent areas.

Here we aim to assess the impact of incorporating socioeconomic metrics in primary care capitation, and total funding through the following key research questions:

1. How accurately does capitation (Carr-Hill) and total funding predict clinical need across measures of morbidity, mortality, and healthcare use?
2. Does adjusting for socioeconomic score and/or age enhance the accuracy of these predictions?

If including socioeconomic status leads to more accurate predictions across a diverse range of clinical need measures, then modifying funding is likely to allocate funding more efficiently according to clinical needs.

## Method

### Study design and data sources

We undertook a neighbourhood-level cross-sectional analysis in England, exploring the inclusion of socioeconomic status in primary care capitation funding. Neighbourhoods were based on Lower layer Super Output Areas (LSOAs), which are 32 844 small geographical areas containing 400–1200 households.^9^ Such analysis is more sensitive to changes in clinical need and funding compared with practice-level analyses.^6^

We used the following health and care data: payments made to each GP practice; the number of registered patients in each practice and within each neighbourhood; the number of weighted patients at each practice following Carr-Hill adjustment; the total number of all appointments made at each practice; disease prevalence data based on practice disease registries; and emergency presentation outcomes for each GP practice.^5^

We used the following demographic data: the annual number of deaths in each neighbourhood per year and the annual age profile of male and females within each neighbourhood.^10^ We used publicly available data on the deprivation score of each LSOA produced by the Office for Health Improvement and Disparities (OHID) using Index of Multiple Deprivation (IMD) scores.^11^ The reporting of this study confers to the Strengthening the Reporting of Observational Studies in Epidemiology (STROBE) statement.^12^

### Variables

Table 1 shows variables and time periods use in the primary analysis.

**Table 1.** Variables utilised in analysis. Further details of the calculation of these is supplied in the Supplementary Information

Variable	Additional details	Time periods utilised in primary analysis
Carr-Hill Index (CHI)	Calculated by the number of weighted patients divided by the number of registered patients for each practice	Financial year 2021–2022
Total general practice payments	Total payments to general practices excluding deductions for pensions, levies, and prescription charge income	Financial year 2021–2022
Median age	Median age of each LSOA	Mid-year 2021
Deprivation score	IMD score for each LSOA	2019
**Outcome variables**		
Combined morbidity index (CMI)	Calculated by the sum prevalence of 18 chronic conditions divided by the number of registered patients for each practice	Financial year 2021–2022
Predicted total morbidity	Includes undiagnosed as well as diagnosed disease. Calculated by the sum prevalence of five chronic conditions divided by the number of registered patients for each practice	2015
Standardised mortality rate (SMR)	Number of deaths in each LSOA in 2022, standardised by age and gender from the reference population of the whole of England in 2022	2022
Emergency department presentations	Number of emergency presentations per 10 000 patients from a general practice’s register	Financial year 2021–2022
Number of GP appointments	Number of total GP appointments per 10 000 patients from a general practice’s register	October, November, and December 2022

IMD = Index of Multiple Deprivation. LSOA = Lower layer Super Output Area

Two measures of funding were included. The CHI of each practice was calculated by dividing the number of Carr-Hill weighted patients by the number of registered patients. This figure is the result of each individual practice’s capitation calculation. Total funding per registered patient to each GP practice, excluding deductions for pensions, levies, and prescription charge income, was obtained. Median age was calculated for each LSOA.

Standardised mortality rates (SMR) were calculated for each LSOA, indirectly age and sex standardised to the population of England. A combined morbidity index (CMI) was calculated for each LSOA, as a strong proxy for LSOA multimorbidity.^6^ To calculate this, in each LSOA, the sum prevalence of 18 chronic condition indicators is divided by the total population.^6^ Additionally, predicted total morbidity was calculated, using data from five indicators available from 2015, which accounted not just for diagnosed but also predicted undiagnosed prevalence. Conditions included in both the CMI, and predicted total morbidity are available in the Supplementary Information. Chosen timescales of all data were selected to ensure that time periods reflected in the dependent variables matched those of the independent variables as much as possible.

General practice data between datasets were linked by practice code. Data from all available practices in all years was used. For any data that required conversion from the level of general practice to LSOA, an average for each LSOA was calculated, weighting for the number of patients in each general practice from each LSOA. Such an approach has been utilised and tested in previous studies, ^6,13^ and is detailed in the Supplementary Information.

### Statistical analysis and reporting

After examining the distribution of the above-mentioned variables, we applied the following transformations to ensure normality: log: total general practice payments, SMR; square root: IMD, emergency department presentations, number of GP appointments; square: predicted total morbidity. To allow comparisons of effect size and magnitudes, all independent variables were standardised (z-scores).

For both measures of funding, and each outcome, we investigated the relationship of dependent variables using four sets of linear models: (1) unadjusted; (2) adjusted for IMD; (3) adjusted for age; and (4) adjusted for age and IMD. Models were weighted for LSOA population. Model performance was assessed by adjusted *R* ^2^ value (adjusted for number of variables), which measures goodness of fit, in this case a measure of each model to effectively measure clinical need. To enable model performance to be judged against out-of-sample performance, while still using all national data, a 10-fold cross validation approach was used. Given that the average age in areas with high IMD is lower (Supplementary Figure 1), adjusting for age was an approach used to address its confounding effect. The magnitude of difference between performance of models 3 and 4 was calculated by the difference of *R* ^2^. *P* values, indicating significance of difference between models, were calculated with analysis of variance (ANOVA).

Coefficients and standard errors were used to calculate the 95% confidence intervals shown in the figures. A significant result was defined as *P*<0.05. All analyses were conducted in R (version 4.3.2).

### Sensitivity analyses

We undertook three sensitivity analyses. Analysis only using pre-pandemic data from 2019 (variables detailed in Supplementary Table 1a). GP appointment numbers were not available for this time; all other outcomes were included. A 3-year average SMR was calculated, compared with main analysis that used data from 1 year only to avoid 2020 death statistics being included. We re-analysed data using 2015 variables because the total predicted mortality data were based on 2015 (Supplementary Table 2). To ensure log transformations of total funding did not bias results, and that a positive skew rather than outliers accounted for large values, analysis was repeated for total funding removing outliers, defined as any result more than 3 SD from the mean (Supplementary Table 3a).

## Results

### Data description

**Figure 1.**
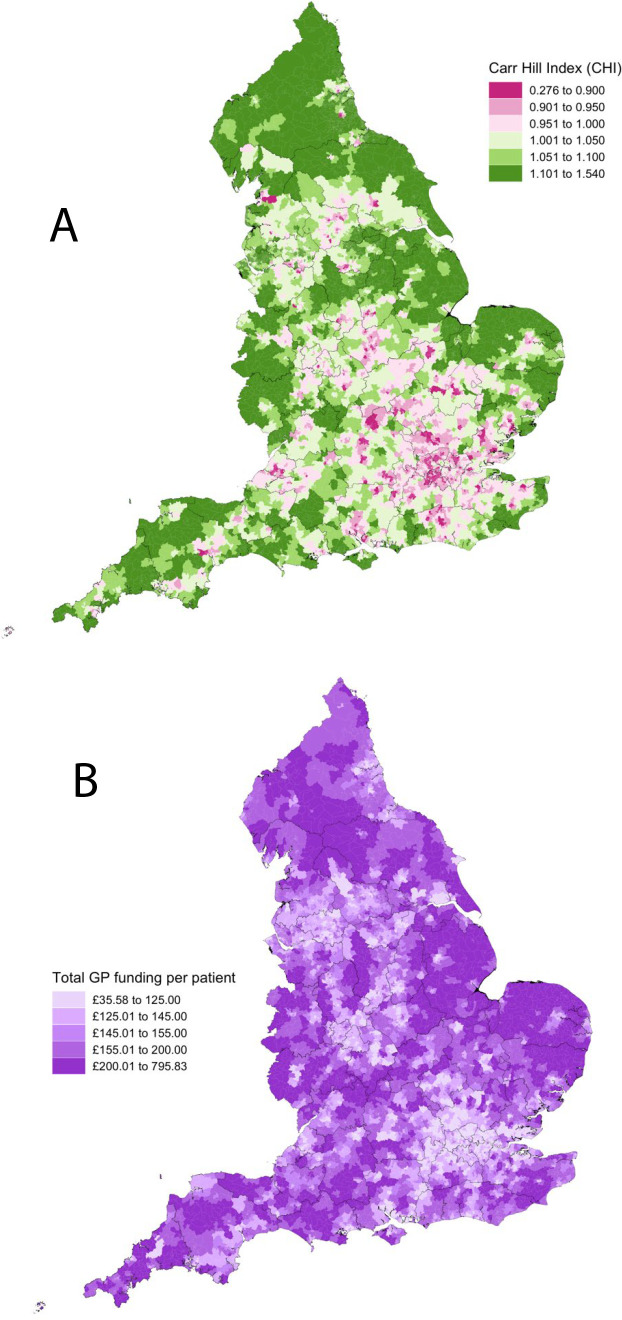
(**A**) Spatial map plotting Carr-Hill Index (CHI) of each Lower layer Super Output Area (LSOA) in England in financial year 2021–2022. Geographical layer shows the 42 integrated care boards in England. (**B**) Spatial map plotting total general practice funding per patient in each LSOA in England in financial year 2021–2022. Geographical layer shows the 42 integrated care boards in England.

Table 2 shows descriptive data, broken down at the level of each of the 32 844 LSOAs. A spatial map of CHI and total funding per person across all of these LSOAs are detailed in Figure 1a and b, respectively.

**Table 2.** Descriptive statistics for each variable

	Mean (SD)	25th percentile	75th percentile
Weighted mean Carr-Hill Index	1.01 (0.09)	0.94	1.06
Total funding per patient	145.71 (36.92)	124.69	155.14
IMD score	21.67 (15.33)	9.91	29.58
Total population	1904.29 (637.72)	1543	2097
Median age	42.16 (7.88)	36.1	48.3
Weighted mean CMI	51.51 (10.79)	45.16	58.68
Weighted mean total predicted morbidity	15.61 (1.84)	14.77	16.7
Standardised mortality rate	1.08 (0.61)	0.69	1.34
ED admissions per 10 000 people	90.56 (31.7)	68.88	110.57
Total GP appointments in LSOA per person	1.45 (0.29)	1.25	1.62

CMI = combined morbidity index. ED = emergency department. IMD = Index of Multiple Deprivation. LSOA = Lower layer Super Output Area

### How accurately do capitation payments predict clinical need and does this improve by including socioeconomic status and/or age?

Table 3 displays the association between CHI, IMD and age with five clinical measures of need. CHI was consistently associated with clinical need (model 1). CHI most accurately predicted CMI (*R* ^2^ = 49.81%), and predicted total morbidity (*R* ^2^ = 43.52%). CHI was moderately predictive of ED admissions (*R* ^2^ = 32.75%) and GP appointments (22.51%), but poorly predicted mortality rates (*R* ^2^ = 0.37%).

**Table 3.** Association of Carr-Hill Index, Index of Multiple Deprivation (IMD) and median age with clinical outcomes

	CMI	Predicted total morbidity	SMR	ED admissions	GP appointments
Model 1					
Carr-Hill Index ß (95% CI)	7.89 (7.8 to 7.97)	35.57 (35.13 to 36.01)	0.03 (0.03 to 0.04)	1.01 (0.99 to 1.02)	5.63 (5.52 to 5.74)
*R* ^2^ model 1	49.81	43.52	0.37	32.75	22.51
Model 2					
Carr-Hill Index ß (95% CI)	8.26 (8.17 to 8.34)	36.00 (35.56 to 36.45)	-0.02 (-0.03 to -0.02)	1.06 (1.04 to 1.08)	5.96 (5.84 to 6.07)
IMD ß (95% CI)	-1.89 (-1.98 to -1.81)	-2.22 (-2.67 to -1.77)	0.27 (0.26 to 0.27)	-0.28 (-0.29 to -0.26)	-1.66 (-1.78 to -1.55)
*R* ^2^ model 2	52.48	43.67	24.66	35.07	24.34
Model 3					
Carr-Hill Index ß (95% CI)	6.03 (5.94 to 6.11)	30.45 (29.98 to 30.92)	0.13 (0.12 to 0.13)	0.77 (0.75 to 0.79)	4.17 (4.05 to 4.29)
Age ß (95% CI)	4.25 (4.17 to 4.34)	11.68 (11.22 to 12.15)	-0.23 (-0.23 to -0.22)	0.54 (0.52 to 0.56)	3.34 (3.22 to 3.46)
*R* ^2^ model 3	61.62	47.35	14.89	40.43	28.99
Model 4					
Carr Hill Index ß (95% CI)	5.49 (5.39 to 5.58)	26.48 (25.94 to 27.03)	0.01 (0.01 to 0.02)	0.73 (0.71 to 0.75)	3.89 (3.75 to 4.03)
Age ß (95% CI)	5 (4.89 to 5.1)	17.16 (16.56 to 17.76)	-0.06 (-0.07 to -0.05)	0.59 (0.57 to 0.61)	3.73 (3.57 to 3.88)
IMD ß (95% CI)	1.09 (0.99 to 1.19)	8.04 (7.48 to 8.61)	0.23 (0.23 to 0.24)	0.08 (0.06 to 0.1)	0.57 (0.42 to 0.71)
*R* ^2^ model 4	62.15	48.57	25.21	40.53	29.11
Improvement following addition of IMD (*R* ^2^ model 4 - *R* ^2^ model 3)	0.53	0.92	10.32	0.10	0.12

Generalised linear model analysis of the association of CHI, median age and IMD on a range of clinical outcomes. ß = beta coefficient for each variable, brackets show 95% confidence intervals. CHI = Carr-Hill Index. CMI = combined morbidity index. ED = emergency department. IMD = Index of Multiple Deprivation. SMR = standardised mortality ratio

Accuracy of all models improved, following adjustment for IMD alone (model 2). However, with the exception of mortality, IMD had a significant negative association for all outcomes. When additionally adjusting for age (model 3), IMD had a significant positive association for all outcomes, indicating that the negative associations seen were owing to the confounding effects of age.

Adjusting capitation for IMD and age (model 4) improved performance of all models compared with adjusting for age alone (model 3). IMD had a significant association in all five outcomes. This difference was largest for mortality (10.32% improvement in *R* ^2^), followed by predicted total mortality (0.92%) and CMI (0.53%). Small improvements were seen for ED admissions (0.10%) and GP appointments (0.12%). This improvement, although small in some models, was strongly significant in all (*P*<0.001).

### How accurately does total funding predict clinical need and does this improve by including socioeconomic status and/or age?

Table 4 shows the association of total funding, age, and IMD with each of the five measures of clinical need. Capitation most accurately predicted CMI (*R* ^2^ = 29.31%), followed by GP appointments (*R* ^2^ = 28.50%), predicted total morbidity (*R* ^2^ = 21.39%) and ED admissions (*R* ^2^ = 16.95%). Total funding poorly predicted mortality rates (*R* ^2^ = 0.42%).

**Table 4.** Association of total funding, IMD and median age with clinical outcomes

	CMI	Predicted total morbidity	SMR	ED admissions	GP appointments
Model 1					
Total funding ß (95% CI)	6.11 (6.01 to 6.21)	25.18 (24.66 to 25.71)	-0.04 (-0.04 to -0.03)	0.73 (0.71 to 0.75)	6.40 (6.29 to 6.51)
*R* ^2^ model 1	29.31	21.39	0.42	16.95	28.50
Model 2					
Total Funding ß (95% CI)	6.11 (6.01 to 6.21)	25.26 (24.74 to 25.78)	-0.03 (-0.04 to -0.03)	0.73 (0.71 to 0.75)	6.39 (6.28 to 6.5)
IMD ß (95% CI)	-0.13 (-0.24 to -0.03)	5.44 (4.91 to 5.96)	0.27 (0.26 to 0.27)	-0.05 (-0.07 to -0.03)	-0.37 (-0.48 to -0.26)
*R* ^2^ model 2	29.32	22.37	24.85	17.04	28.59
Model 3					
Total funding ß (95% CI)	3.78 (3.68 to 3.88)	17.46 (16.92 to 18.01)	0.05 (0.04 to 0.05)	0.42 (0.41 to 0.44)	5.08 (4.97 to 5.2)
Age ß (95% CI)	5.23 (5.14 to 5.33)	17.36 (16.82 to 17.9)	-0.19 (-0.2 to -0.19)	0.69 (0.67 to 0.71)	2.95 (2.84 to 3.07)
*R* ^2^ model 3	47.15	29.82	10.80	29.5	33.54
Model 4					
Total funding ß (95% CI)	3.00 (2.91 to 3.1)	12.94 (12.41 to 13.47)	-0.01 (-0.02 to -0.01)	0.33 (0.31 to 0.35)	4.74 (4.62 to 4.86)
Age ß (95% CI)	7.09 (6.99 to 7.2)	28.13 (27.54 to 28.73)	-0.04 (-0.05 to -0.04)	0.91 (0.89 to 0.93)	3.78 (3.64 to 3.91)
IMD ß (95% CI)	3.27 (3.17 to 3.37)	18.93 (18.38 to 19.47)	0.24 (0.24 to 0.25)	0.38 (0.36 to 0.4)	1.44 (1.32 to 1.57)
*R* ^2^ model 4	53.15	38.47	25.23	32.84	34.58
Improvement following addition of IMD (*R* ^2^ model 4 - *R* ^2^ model 3)	6.00	7.11	14.42	3.34	1.04

Generalised linear model analysis of the association of total funding, median age and IMD on a range of clinical outcomes. ß = beta coefficient for each variable, brackets show 95% confidence intervals. CHI = Carr-Hill Index. ED = emergency department. IMD = Index of Multiple Deprivation

As for capitation, *R* ^2^ of all models improved following adjustment for IMD alone (model 2). IMD was significant in all models, and had a negative association for CMI, ED admissions, and GP appointments. For all outcomes, the effect size of IMD became positive, or significantly more positive when additionally adjusting for age (model 3), again indicating a confounding effect of age on IMD.

Adjusting total funding for IMD and age (model 4) improved performance of all models compared with adjusting for age alone (model 3). Differences were larger than for capitation. This difference was largest for mortality (14.42% improvement in *R* ^2^), followed by predicted total mortality (7.11%), CMI (6.00%), ED admissions (3.34%), and GP appointments (1.04%).

### Sensitivity analysis

Results were robust to a range of sensitivity analyses. Models using pre-pandemic data showed consistent and positive associations between socioeconomic status and clinical need, and inclusion of socioeconomic status models improved performance (Supplementary Tables 1b, 1c). The degree of variation explained by total funding was less than that by capitation. Using predicted total mortality as a single outcome and using independent variables from 2015 returned robust results (Supplementary Table 2b). Results were robust to repeat analysis, when rather than log transforming total funding, a SD cut off was instead applied (Supplementary Table 3).

## Discussion

### Summary

Generally, capitation and total funding were stronger predictors of morbidity, moderate predictors of ED admissions and total GP appointments, and weak predictors of mortality.

IMD was significantly associated with all clinical outcomes, and including IMD enhanced accuracy in all models. This finding was affected by a strong confounding effect of age: when not accounting for age, practices in more deprived areas had artificially lower clinical need because of the younger demographic. For total funding, this effect was seen in three of five outcomes (all but mortality, and predictive total morbidity). When adjusting for age, there was significantly higher clinical need in areas with higher IMD scores for all outcomes, for both measures of funding. Performance of all age and IMD co-adjusted models improved compared with age-adjusted models alone, indicating that adjusting for IMD results in a more accurate measure of clinical need. This effect was larger for total funding, compared with capitation funding.

### Strengths and limitations

The study draws on a vast patient pool, encompassing all registered patients living in England across multiple time frames. It scrutinises the impact of variables at the most detailed neighbourhood level, enhancing the precision and sensitivity of the analyses. Results were sensitive to a robust set of sensitivity analyses, including analyses at different points in time. Analysis was made more robust by the use of adjusted, rather than unadjusted *R* ^2^ value, to account for the effects of increasing the number of variables. The measure of *R* ^2^ value being based on an out of sample prediction significantly increases the reliability of findings and subsequent policy recommendations.

Not all GP practices are funded through the same capitation models. As of 2022, 3.5% of general practices had Alternative Provider Medical Services contracts which do not directly use the Carr-Hill funding to calculate their funding.^5^ However, including the CHI of these practices prevented bias being introduced by geographical variation in contract types.

Measuring by LSOAs allows a detailed, and granular assessment across the country. There are some drawbacks, which have been minimised whenever possible. Assigning practice-level statistics to the level of neighbourhoods introduces the risk of ecological fallacy; for example, as morbidity burden may not be uniform across a practice’s population. However, the risk of this was found to be low in a previous study that also used such analysis.^6^ Second, SMR can be imprecise at the LSOA area. Given this, sensitivity analysis used SMR data over 3 years, which reduced imprecision. Third, the use of LSOAs can increase spatial autocorrelation. However, we consider the risk of this biasing results low given that a previous study using a similar methodology found that spatial autocorrelation in CMI, IMD and funding of LSOAs to be low. Results suggested that this should not have affected the precision of model estimates.^6^

Limitations exist in clinical outcomes utilised. Each outcome is based on factors that are positively associated with increasing age. Given that clinical need is also taken up in early life, further analysis could explore clinical outcomes in the young.

Given cross-sectional analysis, causal effects of funding on clinical outcomes may impact clinical need. This effect would have the most impact on the number of GP appointments, as it represents a short-term measure specific to local GPs. This could explain why the model performance improved when total funding was used as a predictor compared with capitation, which was observed to be more accurate in all other outcome measures.

### Comparison with existing literature

These findings are consistent with other studies, which have found increased clinical need, and worse clinical outcomes in areas with greater IMD score.^14–17^ These results provide further evidence concerning IMD’s association with a diverse range of clinical outcomes at the neighbourhood level, offering further data in the existing evidence base, which does not have sufficient data available at this accurate level.^18^

Compared with a previous study that assessed the association between total GP funding and a range of factors including age, sex, ethnicity, CMI, and IMD at a neighbourhood level, this study achieved better goodness of fit (*R* ^2^ = 62.15% in this study, compared with 39%).^6^ This improvement is likely owing to more up-to-date data, and the use of total funding as an independent rather than outcome variable.

### Implications for research and practice

Much debate exists regarding funding of primary care in England. These results support the inclusion of socioeconomic status in the capitation formula.

Our data suggests that by including socioeconomic status in the capitation formula, funding efficiency is enhanced. Historical evidence underscores this point: from 2002, the UK government significantly improved the economic efficiency of increased funding by directing these resources to more deprived areas.^19^

Data from IMD-adjusted models offer important reflections for any future policy. For seven of ten outcomes (Table 3 model 3, Table 4 model 3), when no adjustment for age was made, the coefficient of socioeconomic status was negative. This effect was seen owing to the confounding effect of age, whereby more affluent areas have older populations. Simply recalculating the Carr-Hill capitation formula with adjustment for IMD alone could therefore result in more funding going to affluent areas owing to their older populations. This would perpetuate existing primary care inequalities.^6,7,17,20^ In order to accurately adjust for socioeconomic status, more robust co-adjustment for age, or alternatively adjusting for better measures of prevention rather than outcomes is needed.

The poor performance of both capitation and total funding to predict long-term mortality, compared with shorter-term outcomes, raises further questions regarding the desired outcome from funding. Increasing primary care funding reduces mortality, especially in areas with greater deprivation scores.^19^ Rather than funding reactively reflecting short-term measures, should policymakers aim to leverage this effect to reduce mortality inequalities, then these findings offer a significant opportunity: funding should better account for differences in mortality.

Models using capitation more accurately predicted clinical need for three of five outcomes. The lower accuracy of total funding to predict clinical need signals inefficiencies within funding and may support larger use of capitation models.^21^ Some evidence suggests reduced efficiency in funding distribution can be caused by funding for prescription charges, Quality and Outcomes Framework (QOF) and pay-for-services schemes.^22–24^ To counter this, such payments could be adjusted for clinical need.

In conclusion, capitation models better reflected clinical need than total funding. Both capitation model and total funding were poor predictors of long-term mortality, indicating a limitation in current funding models. These findings offer the strongest evidence to date that adjusting both capitation payments and total funding by socioeconomic status would result in a more accurate prediction of clinical need.

## Data

Data available upon reasonable request.
